# Optimal Management of the Critically Ill: Anaesthesia, Monitoring, Data Capture, and Point-of-Care Technological Practices in Ovine Models of Critical Care

**DOI:** 10.1155/2014/468309

**Published:** 2014-03-25

**Authors:** Saul Chemonges, Kiran Shekar, John-Paul Tung, Kimble R. Dunster, Sara Diab, David Platts, Ryan P. Watts, Shaun D. Gregory, Samuel Foley, Gabriela Simonova, Charles McDonald, Rylan Hayes, Judith Bellpart, Daniel Timms, Michelle Chew, Yoke L. Fung, Michael Toon, Marc O. Maybauer, John F. Fraser

**Affiliations:** ^1^Critical Care Research Group Laboratory, The Prince Charles Hospital, Rode Road, Chermside, Brisbane, QLD 4032, Australia; ^2^The University of Queensland, St Lucia, Brisbane, QLD 4072, Australia; ^3^Medical Engineering Research Facility (MERF), Queensland University of Technology, Brisbane, QLD 4001, Australia; ^4^Bond University, Gold Coast, QLD 4226, Australia; ^5^Research and Development, Australian Red Cross Blood Service, Kelvin Grove, Brisbane, QLD 4059, Australia; ^6^Science and Engineering Faculty, Queensland University of Technology, Brisbane, QLD 4001, Australia; ^7^Department of Emergency Medicine, Princess Alexandra Hospital, 199 Ipswich Road, Woolloongabba, QLD 4102, Australia; ^8^Innovative Cardiovascular Engineering and Technology Laboratory, The Prince Charles Hospital, Chermside, Brisbane, QLD 4032, Australia

## Abstract

Animal models of critical illness are vital in biomedical research. They provide possibilities for the investigation of pathophysiological processes that may not otherwise be possible in humans. In order to be clinically applicable, the model should simulate the critical care situation realistically, including anaesthesia, monitoring, sampling, utilising appropriate personnel skill mix, and therapeutic interventions. There are limited data documenting the constitution of ideal technologically advanced large animal critical care practices and all the processes of the animal model. In this paper, we describe the procedure of animal preparation, anaesthesia induction and maintenance, physiologic monitoring, data capture, point-of-care technology, and animal aftercare that has been successfully used to study several novel ovine models of critical illness. The relevant investigations are on respiratory failure due to smoke inhalation, transfusion related acute lung injury, endotoxin-induced proteogenomic alterations, haemorrhagic shock, septic shock, brain death, cerebral microcirculation, and artificial heart studies. We have demonstrated the functionality of monitoring practices during anaesthesia required to provide a platform for undertaking systematic investigations in complex ovine models of critical illness.

## 1. Introduction 

Optimal management of the critically ill is the goal for ongoing research utilising clinically relevant animal models. The body size and close similarities between sheep and human pulmonary anatomy, physiology, and immunology make sheep a suitable species for studying pathologies afflicting humans [1–3]. In comparison, swine also offer considerable advantages over other large animal models in biomedical research for the same or more reasons as sheep and ease of management [4–6]. Anaesthesia is a requirement during interventions in animal research to minimise or eliminate the experimental induction of pain. Optimal anaesthesia and analgesia need to be titrated to the individual animal under close monitoring and supervision aided by the use of patient monitors, data capturing devices, and patient point-of-care technology. Patient monitors are designed to aid in determining priorities for care and are not meant to replace the clinician [7–9] at the tableside. They provide an avenue for recording significant events to enhance the recognition of trends in important physiological variables [9–12]. Complex experiments using ovine models therefore necessitate access to a well-equipped theatre with advanced organ system monitoring and point-of-care technology that allows real-time tailoring of therapy and standardisation of anaesthesia and critical care practices. Additionally, appropriately trained researchers with a thorough understanding of the use and interpretation of these systems and of ovine physiology are essential.

Currently, there are varied methodologies of ovine anaesthesia, monitoring, organ support, and point-of-care devices in use in large animal intensive care units (ICUs) and research facilities around the world. To date, however, there are limited studies that describe in detail sheep anaesthesia and monitoring practices in biomedical research establishments with modern large animal ICUs. The aim of this paper is to provide an overview of anaesthesia and monitoring practices used in sheep in comparison with a detailed description of the procedure of animal preparation, anaesthesia and monitoring, critical care, data capture, and point-of-care technology developed by our research group on several validated ovine models (Table 1). It is expected that this paper will provide a foundation for comparison of the available ICU monitoring technology for large animal experimental models and will set an example of recognising the available anaesthetics and standards of devices currently marketed for use in large animal models of intensive care.

## 2. Overview of Anaesthesia and Monitoring Practices for Ovine Models

Reports from the past ten years show that there were considerable variations in anaesthesia and monitoring practices in sheep utilised in biomedical research [2, 17, 29–76]. Ketamine was the most commonly used induction agent followed by thiopentone. Only a few studies used alfaxalone for induction of anaesthesia [2, 17, 31, 76]. For maintenance of anaesthesia, halothane in O_2_ was the most commonly used agent followed by isoflurane in O_2_. A limited number of studies used alfaxalone for maintenance of anaesthesia [2, 17, 76]. Female sheep of the Merino breed were used in most of the experiments probably because of their docile nature and relative availability compared to males. While it is difficult to parse out the rationale for the various alternative approaches for ovine anaesthesia and monitoring in many published studies, the experimental goal, at least in part, impacts the procedure to be implemented. Several studies required anaesthesia only during instrumentation, as subsequent experimental procedures required sheep to be conscious. In sheep that required assisted ventilation, the Servo ventilator (model 900C, Siemens-Elena; Solna, Sweden) was the most commonly used device. In studies that documented the method of euthanasia of sheep, the administration of ketamine anaesthesia in combination with a saturated potassium chloride solution was the most commonly used approach. Unlike companion and other laboratory animal anaesthesia, small ruminant anaesthesia is an area that is still evolving, which may explain the variability in anaesthesia and monitoring techniques. Furthermore, sheep are not routinely anaesthetised in veterinary practice; therefore, few anaesthetics or procedures have been developed specifically for this species. For this reason and probably because they are food animals, many anaesthetic drugs are not registered for use in sheep. Several other factors that may affect the choice of anaesthesia and monitoring include experience with the species, anaesthetic drug costs and availability, and monitoring facilities.

There is also considerable diversity in the types of technological devices used for physiological monitoring during anaesthesia and equipment used in point-of-care in various centres that have studied sheep. Some differences of these devices are notably in the type of physiological monitors, blood gas analysers, pressure transducers, anaesthetic gas analysers, blood flow meters, data acquisition recorders, oximeters, cardiac output monitors, brain monitors, blood cell counters, protein assayers, and colloid osmometers (Table 2).

A consideration of all the factors on anaesthetics and monitoring technology is very helpful for aiding future investigators in making the appropriate choices for the sort of gear required for procedures they would like to implement in their large animal studies.

## 3. Laboratory Practices for Ovine Models at the Critical Care Research Group (CCRG) in Australia

In this section, we describe anaesthesia, critical care and monitoring practices, data capture, and point-of-care technological techniques used at a large Australian animal research laboratory. The CCRG Laboratory has considerable experience in anaesthetising sheep under ICU conditions and has developed several validated ovine models. The areas of discussion are classified into anaesthesia practice, advanced monitoring, auxiliary life support equipment, and point-of-care technology. Brief overviews of the relevant ovine models are also provided.

### 3.1. Animal Care, Anaesthesia, and Monitoring

Animals are treated in accordance with the Australian Code of Practice for the Care and Use of Animals for Scientific Purposes [77]. All studies are registered with institutional Animal Welfare and Ethics departments and have the Animal Ethics Committee (AEC) approval from The Queensland University of Technology (QUT) and The University of Queensland (UQ). Local guidelines mandate the presence of at least two trained personnel to be with the animal model at all times for the duration of a given experiment. This is necessary not only for animal welfare purposes but also for the proactive minimisation of staff fatigue. A team of scientists, nurses, veterinarians, intensive care specialists, surgeons, cardiologists, and perfusionists are present during commencement of complex experiments and remain on call for the duration of the experiment. If at any time the animal becomes physiologically distressed to such an extent that it cannot be managed or reversed, it will be immediately euthanized and documented accordingly.

#### 3.1.1. Animal Selection and Preanaesthetic Care

Batches of healthy adult sheep* (Ovis aries)* are procured from a breeding institution and reared as a flock in an open pasture farm. Sheep sizes are tailored to specific experiments and their weights are generally in the range of that of a small adult human [78]. Within two weeks prior to experiments, animals are transported to and housed at a purpose built animal facility and managed as per standard operating procedures. The sheep are fed with proprietary sheep feed and lucerne and have free access to water. Shelter is provided in built sheds in which the sheep have to free access. Shade is provided by large trees in the paddocks and the sheep interact freely with each other. In general, animals are fasted overnight with free access to drinking water until two hours before the procedure. The sheep are subjected to preexperimental complete veterinary clinical examination which includes body temperature, pulse, and respiratory (TPR) parameter checks. The sheep are then restrained in a sling cage and the ventral aspect of the neck is shaved to facilitate vascular access.

#### 3.1.2. Anaesthesia Technique

**Figure 1:**
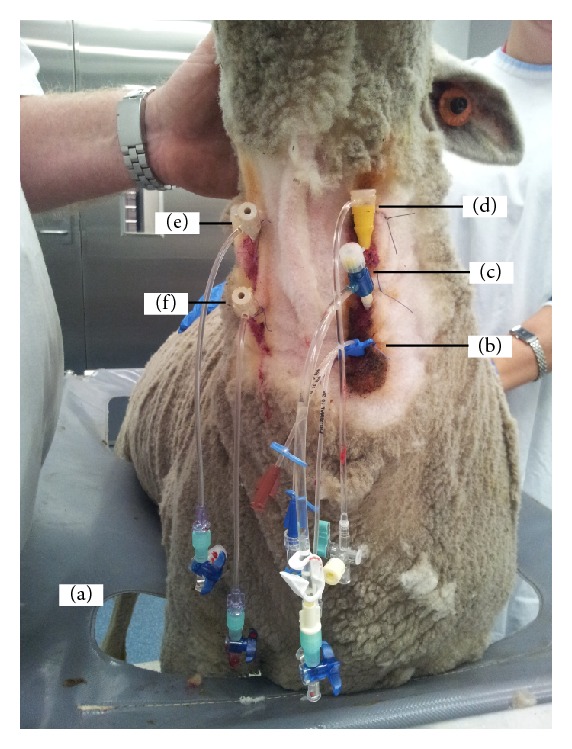
An adult Merino ewe being prepared for venovenous extracorporeal membrane oxygenation (VV-ECMO). VV-ECMO is implemented in patients with severe respiratory failure refractory to conventional ventilatory support to provide gas exchange. Venous blood from the patient is accessed from large central veins and returned to the right atrium after it has passed through an oxygenator. The animal has been restrained in a sling cage (a); ventral neck hair has been clipped and aseptically prepared to allow intravenous access to be gained; note the brown colour of povidone iodine. A multilumen central venous catheter has been inserted into the left jugular vein of the animal under local anaesthetic and sutured into place (b) to allow for blood sampling and medications and fluid administration. The left jugular vein has been further cannulated with an 8G sheath for the insertion of a pulmonary artery catheter (c) for haemodynamic monitoring. An 11G sheath catheter has been inserted proximally into the left jugular vein under local anaesthetic and sutured into place to allow for intracardiac echocardiography (ICE) catheter to be inserted (d). The right jugular vein has been cannulated both proximally (e) and distally (f) with single lumen central lines to aid subsequent insertion of return and access ECMO cannulae, respectively. Incremental doses of midazolam are administered to maintain sheep comfort during the prolonged procedure.

Each study has a different protocol of maintenance drugs for total intravenous anaesthesia (TIVA), but the initial induction and instrumentation for haemodynamic monitoring is essentially identical across all the studies. The BSD studies form the foundation of this technique [27]. Under local anaesthesia with lignocaine 1%, a 7FG triple-lumen central venous line (Arrow-Howes, Research Triangle Park, NC, USA) is placed in the external jugular vein (EJV) of the awake sheep. The internal jugular vein (IJV) is usually of small calibre and is often absent in sheep [80]. This catheter is used for initial blood sampling and for intravenous (IV) administration of premedication, induction agents, drugs, fluids, and TIVA throughout the procedure. Following any required baseline blood sampling, the sheep are administered buprenorphine (0.01 mg/kg) by slow IV injection to minimise distress during subsequent procedures. For experiments requiring prolonged cannulation procedures, further sedation with midazolam is indicated. An 8Fr sheath is placed in the left EJV under local anaesthesia for subsequent placement of a pulmonary artery catheter (Edwards Lifesciences, Irvine, CA, USA). Additional sheaths may be placed in the opposite EJV to facilitate cannulation and intracardiac echocardiography (ICE) in ovine extracorporeal membrane oxygenation (ECMO) experiments (Figure 1). General anaesthesia is induced by intravenous administration of midazolam (0.5 mg/kg) and alfaxalone (3 mg/kg) (Alfaxan Anaesthetic Injection, Jurox Pty Ltd., NSW, Australia). Alfaxalone is given in 4 aliquots at 15 s intervals. Anaesthesia is maintained with continuous infusions of alfaxalone (4–6 mg/kg/hr), ketamine (3–5 mg/kg/hr), and midazolam (0.25–0.5 mg/kg/hr). Buprenorphine 0.01 mg/kg is administered intravenously every six hours throughout the study period for ongoing analgesia. Anaesthetic drug doses are titrated to maintain an appropriate plane of anaesthesia determined by monitoring heart rate, blood pressure, respiratory rate, eyelash reflexes, chewing, jaw movements, and haemodynamics [81–84]. Gentamicin 240 mg and cephalothin 1 g are administered intravenously for presurgical prophylaxis in lengthy experiments such 24 h ECMO studies.

Alfaxalone is currently only approved for use in cats and dogs [85–88]. Animal models such as those that are being developed for investigation of critical illness result in significant physiological insult to the experimental subject. Hypoxia, acidosis, and haemodynamic instability are common sequelae of the experimental pathological states under investigation. Consequently, the choice of the anaesthetic agent is guided by effective anaesthesia and analgesia and appropriate surgical conditions. Haemodynamic stability is an essential consideration. Agents such as inhaled anaesthetics or propofol often cause hypotension, which could interfere with the effect of the insult under investigation. Alfaxalone provides consistent anaesthetic conditions with good haemodynamic stability [89]. It however continues to be considerably expensive, leading to significant limitations for its use in large animals. Addition of ketamine and midazolam provides analgesia as well as reducing the requirement of alfaxalone as a single agent for anaesthesia. To our knowledge, it is only our studies that have used and continue to use alfaxalone for induction and maintenance of anaesthesia in a large number of sheep. We have been very successful with the combination of alfaxalone, ketamine, midazolam, and buprenorphine with or without fentanyl for the maintenance of anaesthesia and analgesia. This approach continues to be the mainstay for TIVA in our experiments with consistent and desirable anaesthetic outcomes.

#### 3.1.3. Airway Access and Ventilation

The sheep are intubated whilst in the sling cage with assistance from animal handlers. Orotracheal intubation is performed with size 8, 9, or 10 cuffed endotracheal tube, depending on sheep size (Portex; Smiths Medical, Australia) under direct laryngoscopy and ventilation with 100% oxygen via an ambu bag. Sheep are then placed on the surgical table (or into a metabolic cage depending on the study) in lateral recumbent position for further instrumentation. Mechanical ventilation is commenced using a Galileo intensive care ventilator (Hamilton Medical AG, Switzerland) with a sidestream end-tidal CO_2_ (ETCO_2_) detector (Marquette TRAM, GE Healthcare, Waukesha, WI, USA). The initial ventilator tidal volume is set to approximately 10 mL/kg with a respiratory rate of 15 breaths/min, positive end expiratory pressure (PEEP) of 5 cm H_2_O, and initial FiO_2_ (fraction of inspired oxygen) of 1.0. These settings are then titrated based on arterial blood gas (ABG) results. In studies involving models of acute lung injury, a low tidal volume, high PEEP strategy is employed to minimise ventilator induced lung injury [90–92]. In the majority of studies, a tracheostomy is performed to facilitate bronchoalveolar sampling. This is necessary as standard bronchoscopes are not of sufficient length to access segmental bronchi in sheep through an endotracheal tube. An appropriately sized tracheostomy tube (average size 7–10) (Portex, Smiths Medical, Australia) is inserted surgically into the trachea via a 3 cm transverse incision made between two tracheal rings at approximately 6 tracheal rings distal to the cricoid cartilage. After securing the airway, the orotracheal tube is then withdrawn and animals are ventilated through the tracheostomy tube. An orogastric tube is inserted and the distal end is sutured to the lower lip and allowed free drainage or suction to prevent ruminal tympany.

#### 3.1.4. Haemodynamic Monitoring

Detailed reviews of principles and practices of haemodynamic monitoring and management are beyond the scope of this paper; readers are referred elsewhere [93–98]. The transfacial artery is surgically exposed and cannulated for invasive blood pressure monitoring and for ABG sampling. Physiologic variables are continually monitored with the Marquette Solar 8000 Patient ICU CCU Monitoring System (GE Healthcare, Waukesha, WI, USA) and recorded every five seconds with custom software. A pulmonary artery catheter (Swan-Ganz CCOmbo, Edwards Lifesciences, Irvine, CA, USA) is inserted via the EJV sheath and is coupled with the Vigilance II monitor (Edwards Lifesciences, Irvine, CA, USA) to record continuous cardiac output (CCO), mixed venous oxygen saturation (SvO_2_), central venous pressure (CVP), pulmonary artery pressure (PAP), systemic vascular resistance (SVR), stroke volume (SV), and core body temperature (BT). Indices can be corrected for body surface area (BSA) using the following equation: BSA (m^2^) = 0.094 (body weight in kg)^0.67^ [99]. Measured variables are recorded every five seconds using proprietary software. CVP and PAP measurements are taken from the respective ports of the Swan-Ganz CCOmbo catheter. Pulmonary artery occlusion pressure (PAOP) can also be measured when required. Direct arterial blood pressure (ABP) measurements are taken from the facial artery and transduced to the Marquette Solar 8000 monitor. Electrocardiography (ECG) readings are achieved via an ECG module and routed to the monitor. The accuracy of any devices such as those used to measure blood pressure is an important consideration when selecting monitoring equipment for use in animals [100]. We routinely measure and monitor direct ABP and CVP. Monitoring of CVP is useful in measurement of hydrostatic pressure in the intrathoracic vena cava which is a useful guide not only in fluid replacement therapy but also to the ability of the right heart to handle the venous return [9]. Direct blood pressure measurement is not without problems; clotting in the catheter can occur; time is required to instrument the patient; the electronic equipment is expensive; and potentially fatal complications can occur [101].

The laboratory is also equipped with an ultrasonic cardiac output monitor (USCOM A1, Sydney, NSW, Australia). This device is used to guide haemodynamic management and to collect additional haemodynamic data [102, 103]. Depending on the experiment, transthoracic, epicardiac, or intracardiac echocardiography may be used in our models for cardiac function assessment and data collection purposes.

#### 3.1.5. Respiratory Monitoring

Continuous pulse oximetry (SPO_2_) and ETCO_2_ recordings are achieved via couplings to the Marquette Solar 8000 monitor. Pulse oximetry provides a measure of oxyhaemoglobin saturation in blood and is one of the most utilised monitoring aids in the critically ill or emergent patient [7, 9, 10, 104]. Accuracy and failure rates (failure to produce a reading) vary widely from model to model and from species to species [105]. We obtain reasonably accurate ETCO_2_ measurements that provide valuable information on the ventilatory status of the patient [9–11, 104, 106]. ETCO_2_ measurement also provides an early warning of serious hypotension and very low cardiac output from the right side of the heart and detects apnoea and breathing circuit disconnections [10, 106]. Arterial blood samples are collected from the facial artery at predetermined intervals depending on the study. ABGs are determined using an automated analyser (ABL800 Flex Radiometer, Copenhagen, Denmark), providing readings for arterial partial pressures of O_2_ (P_a_O_2_) and carbon dioxide (P_a_CO_2_). The ABG analyser also provides absolute values for oximetry including the concentrations of total blood haemoglobin (ctHb), oxygen saturation (sO_2_), fractions of oxyhaemoglobin (FO_2_Hb), carboxyhaemoglobin (FCOHb), methaemoglobin (FMetHb), deoxyhaemoglobin (FHHb), and foetal haemoglobin (FHbF). The ABG analyser is used to monitor blood acid-base status (pH), electrolytes (chloride, calcium, potassium, and sodium ions), and metabolites (blood glucose and lactate). Ventilation data are recorded on a breath-by-breath basis using inbuilt software of the Galileo ventilator. Readings of pulmonary static compliance are recorded from the ventilator at predetermined intervals.

In some animal models, electrical impedance tomography (EIT) is used to determine the spatial impedance distribution in a body cross-section by providing a dynamic, breath-to-breath measurement of both global and regional ventilation [107, 108]. EIT monitoring has the potential to become a noninvasive bedside tool for assessment of regional lung ventilation and lung recruitment in patients with acute respiratory failure [107–112].

#### 3.1.6. Microcirculation and Tissue Monitoring

Concomitant monitoring of the macro- and the microcirculation using sidestream dark-field (SDF) imaging is often necessary in studies investigating shock and mechanisms of organ injury. For example, in the studies that examine the tissue effects of resuscitation with various fluids and blood products in ovine models of haemorrhage, trauma, and sepsis, real-time tissue oxygenation [113–116] is monitored by the insertion of oxygen probes (OxyFlow, Optronix, Oxford, UK) into the brain, heart, renal cortex, liver, and skeletal muscle. Tissue perfusion [113–116] is monitored by the insertion of Laser Doppler Flow probes (MNP100XP or MNP110XP, OxyFlow, Optronix, Oxford, UK) into the brain, heart, renal cortex, liver, and skeletal muscle. Tissue metabolism is monitored by the insertion of microdialysis [117–121] catheters into the anterior surface of the left ventricle, renal cortex, liver, skeletal muscle, and the femoral artery. These monitoring techniques are technically demanding; therefore, standardisation of anaesthesia, resuscitation and haemodynamic support practices, and strict implementation of study protocol are crucial to procuring quality data. Details of available techniques to monitor the microcirculation are beyond the scope of this paper and can be found elsewhere [113–116].

#### 3.1.7. Temperature, Fluid, Vasoactive Drugs, and Electrolyte Management

Continuous temperature recording is important in order to detect hypo- or hyperthermia in a timely manner and institute corrective measures [122]. Animal patients lose body heat very rapidly when anaesthetised and precautions should be taken to avoid this [122, 123]. Temperature is managed using a circulating warm water mattress attached to either the warm water pump cooler or heater (Hemotherm, Cincinnati, OH, USA). Maintenance fluids (compound sodium lactate, Plasma-Lyte 148 Replacement, dextrose solutions, dextrose/saline solutions, or 0.9% sodium chloride solution) are delivered intravenously at 38-39°C and generally run throughout studies at a rate of 2 mL/kg/hr or at 10 mL/kg/hr during surgical interventions. Additional fluid boluses may be required to maintain blood pressure (BP) and CO as both BP and CO are clinically relevant to achieve mean arterial pressure (MAP) > 65 mmHg and CO > 3.0 L/min. Dopamine infusion (1 mg/mL preparation) titrated to effect is utilised as an inotrope if there is evidence of tissue hypoperfusion despite the fact that normal or elevated SVR occurs. Noradrenaline infusion (60 *μ*g/mL) is commenced and titrated to effect if there is a decrease in BP, +/− a decrease in CO, and a decrease in SVR. These haemodynamic parameters are a rough guide only and may vary between experiments depending on the research question for a given study. In our experience with vasoactive drugs in sheep, glyceryl trinitrate is associated with poor outcomes. Dobutamine causes profound tachycardia. Adrenaline is given in very minute doses if required. Hydralazine and sodium nitroprusside are great in decreasing afterload. Amiodarone when indicated in the management of dysrhythmias is given in minute doses by slow infusion as sheep are very prone to heart block. Maintenance fluids are changed to 5% dextrose in normal saline if blood glucose drops below 2 mmol/L. Potassium chloride solution infusion is given to achieve measured K^+^ of 3–5 mmol/L at a titrated rate of 5–20 mmol/hr as needed. In case of persistent dysrhythmias, 20 mmol of magnesium sulphate solution is given over 30 min. Calcium chloride makes sheep extremely tachycardiac; therefore, we give calcium gluconate in 2.2 mmol boluses to achieve a measured Ca^2+^ above 1 mmol/L. Bicarbonate levels are corrected with sodium bicarbonate infusion if the animal is critically ill and acidotic depending on the bicarbonate deficiency.

#### 3.1.8. Ovine Blood Collection, Storage, and Transfusion

Healthy adult sheep permit the collection of a unit of whole blood (~400 mL) into standard human blood collection bags [20, 124]. Blood is collected through a venous sheath in the EJV into Leukotrap WB blood bags (Pall Medical UK), incubated, leukofiltered, and manually separated into plasma and packed red blood cells (PRBCs) using a plasma extractor (Fenwal, Baxter, USA). The PRBCs are resuspended in saline-adenine-glucose-mannitol (SAG-M) additive solution and stored at 2–6°C, while plasma is frozen at −20°C [124]. Human cross-matching protocols have been modified for sheep, and our observations have detected 18.2% incompatibility between ovine PRBCs (ovPRBCs) and potential recipient sheep [20, 125]. This highlights the importance of cross-matching especially in transfusion models [20, 124].

#### 3.1.9. Sample Collection and Processing

An indwelling Foley urinary catheter is inserted urethrally in ewes and attached to a urine collection bag for measurement of urinary output and to facilitate urine sampling for urinalysis. Blood samples are taken via the facial arterial line or central venous line only and processed for submission to the laboratory or instantly for blood gas analysis. Bronchoalveolar lavage (BAL) and exhaled breath condensate samples are taken at time points determined by the individual studies.

#### 3.1.10. Euthanasia Technique

At completion of experiments, animals are euthanized with intravenous administration of sodium pentobarbitone 325 mg/mL (Lethabarb, Virbac, Australia) at 0.5 mL/kg. Death of the animal is confirmed by the loss of cardiac electrical activity, blood pressure, cardiac output, and reflex activity.

#### 3.1.11. Postmortem Handling and Disposal

A mid-ventral sternotomy or lateral thoracotomy (depending on the model) and coeliotomy are performed. The organs of interest are examined* in situ* before being harvested. Tissue samples for gene expression analysis are collected in RNAlater solution (Life Technologies, Thermo Fisher Scientific Inc.) or snap frozen in liquid nitrogen before being stored at −80°C. Histological samples are taken and fixed in formalin for later processing and staining. The rest of the animal remains are frozen and stored until being able to be disposed of via high temperature incineration.

### 3.2. Animal ICU Equipment and Point-of-Care Technology

**Figure 2:**
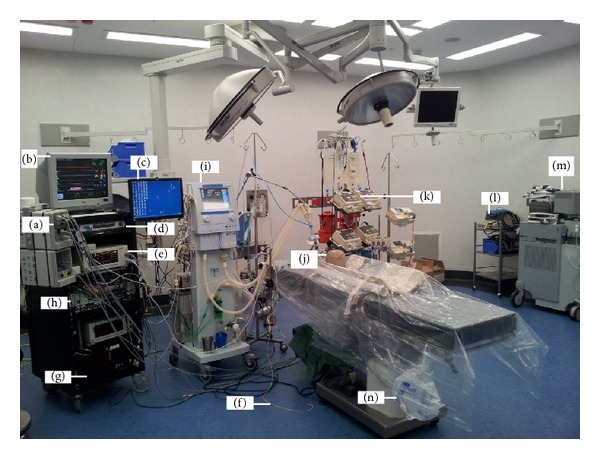
Standard animal intensive care unit (ICU) theatre of the Critical Care Research Group (CCRG). For continuous haemodynamic monitoring, the Marquette Solar 8000 Patient ICU CCU Monitoring System (GE Healthcare, Waukesha, WI, USA) consisting of modules (a), displays (b and c), and processor unit (d) together with Vigilance II monitor (e) coupled with Swan-Ganz CCOmbo pulmonary artery catheter (f) (Edwards Lifesciences, Irvine, CA, USA) are used. Other accessories include the hard disc (g) for data storage and an analogue to digital data converter (h). Recruitment of the intensive care ventilator Galileo (Hamilton Medical AG, Switzerland) (i) is performed with the aid of an Ohmeda breathing bag (j) before every experiment. Fluids and medications are delivered from the intravenous fluid workstation (k). Some experiments require the use of an electrosurgical unit (l) and echocardiography (m). All animals have bladder catheters and urine is collected in a sterile bag (n).

All studies use the same standard setup for advanced haemodynamic monitoring, mechanical ventilation, and fluid therapy (Table 3). Individual studies may require additional sets of equipment and operating table preparation.  Figure 2 depicts the theatre setup for ECMO studies.

An intensive care ventilator (Galileo, Hamilton Medical AG, Switzerland) is utilised for mechanical ventilation. This advanced microprocessor controlled intensive care ventilator offers a full spectrum of capabilities, including invasive, noninvasive, and advanced ventilation modes plus tube resistance compensation. It also allows inhalational delivery of drugs. The expiratory gas condensate samples are collected via an accessory attachment for a double surface condenser attached to a chiller (Digital Temperature Controller model 9102, PolyScience, IL, USA). Contemporary ventilators are versatile in that they have multiple modes of pressure or volume controlled cycles. Ventilator terminology, settings, patient setup, monitoring, and some of the common complications associated with mechanical ventilation are beyond the scope of this paper and readers are referred elsewhere [126]. In the era of lung-protective mechanical ventilation using limited tidal volumes, higher respiratory rates are applied to maintain adequate minute volume ventilation. Patient-ventilator dyssynchrony can be a problem during mechanical ventilation and can lead to patient discomfort and an increased work of breathing [126–132]. To minimise patient-ventilator dyssynchrony, the sheep are ventilated to achieve ETCO_2_ below 30 mmHg, which inhibits the spontaneous respiratory drive, therefore avoiding the use of neuromuscular blocking drugs. Proper interpretation of ventilator waveforms affords the critical care clinician a better understanding of the patient's respiratory function, response to therapy, and causes of patient-ventilator dyssynchrony [132]. The ventilator also acts as a ventilation meter, respiratory rate monitor, and apnoea alarm.

The ABL800 Flex (Radiometer, Copenhagen, Denmark) analyser is used for blood gas analysis. This analyser measures any combination of pH, blood gas, electrolyte, oximetry, and metabolite parameters. It can measure up to 18 parameters on the same blood sample. The refrigerated Sigma 2-16PK (Sigma, Germany) centrifuge is used for spinning blood and BAL samples for submission to the laboratory. This centrifuge has the microhaematocrit spinning capabilities for point-of-care measurement and monitoring of packed cell volume (PCV). A clinical veterinary refractometer is used for measuring and monitoring plasma protein and urine specific gravity. A bronchoscope (Olympus) is used for examining the trachea and bronchi and for bronchoalveolar lavage. A large animal laryngoscope is used to facilitate endotracheal intubation. We utilise the mobile C-arm X-ray generator with a flat detector (Veradius Neo, Royal Philips Electronics, The Netherlands) for radiography or fluoroscopy to verify placement and migration of invasive catheters and for experimental data collection as required.

Tissue chemistry analysis is accomplished with patient-side microdialysis equipment which includes the microdialysis pumps CMA 107 and CMA/102 (CMA Microdialysis AB, Solna, Sweden) and the point-of-care microdialysis analyser (ISCUSflex, M Dialysis AB, Solna, Sweden). The ISCUSflex analyser takes measurements to monitor tissue chemistry from within microdialysis samples taken from virtually any tissue or organ in the body. Up to six different metabolites (glucose, lactate, pyruvate, glycerol, glutamate, and urea are assayed) provide unique opportunities for early detection of metabolic crisis and ischemia and for guiding therapeutic interventions. Tissue oxygenation and blood flow monitors are used for continuous monitoring. These machines permit continuous and simultaneous measurement of tissue oxygenation, blood flow, and temperature from the same probe or tissue microregion.

In addition to the traditional measurement of activated clotting time (ACT) of whole blood at point-of-care with a cage-side blood clotting system (Hemochron, ITC, NJ, USA), there are two pieces of equipment for advanced coagulation studies. These include the rotating pin thromboelastometry (ROTEM, Tem International GmbH, Munich, Germany) and the multiple platelet function analyser (Multiplate, Verum Diagnostica GmbH, Munich, Germany). The ROTEM uses thromboelastometry to determine if haemorrhage is caused by surgical bleeding, platelet dysfunction, clotting factor insufficiency, fibrin insufficiency, or hyperfibrinolysis [133–138]. The Multiplate uses impedance aggregometry to assess platelet function in whole blood [139–143].

### 3.3. Data Capture and Archiving

We have developed novel techniques for electronic data collection and extraction. Digital data are collected and saved in a computer hard drive from the Marquette monitor as 5-second snapshots of the current values of the variables, the ventilator as breath-by-breath, the ECMO pump at 5-second intervals, and haemodynamic data by the Vigilance II at 2-second intervals. Analogue signals, if available, are routed into custom written software and recorded at an appropriate frequency, often 200 Hz or 1 kHz. During data collection, if the original signals are not optimum, for instance, a poor ECG, a damped ABP, or the oximeter probe is not attached, then the extracted data will reflect this as either missing data or incorrect data. Human error may also play a role especially due to fatigue or unfamiliarity with patient monitors. The data acquisition programme may crash and may not restart soon enough for continuity of the data.

Data are extracted in text form from the files quasi-automatically using an assortment of purpose written programmes and macros into proprietary computer spread sheets. Each study is in a directory based on the date and time. Automated extraction, albeit practical, can lead to some glitches; for instance, if a variable is missing or inaccurate at an exact point, it will be extracted as it is. Data once captured is prospectively analysed to ensure integrity and quality.

### 3.4. Synopses of Validated Ovine Models and Related Studies

#### 3.4.1. ECMO Studies

**Figure 3:**
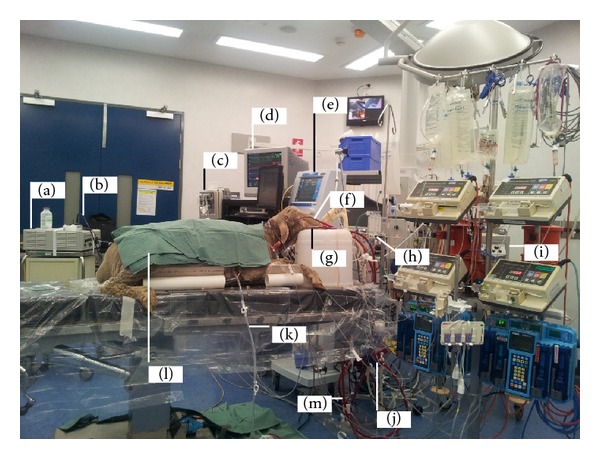
Sheep on venovenous extracorporeal membrane oxygenation (VV-ECMO). Equipment includes the Stryker light source (a) attached to an Olympus bronchoscope (b) for bronchoscopy and bronchoalveolar lavage. The Marquette Solar 8000 Patient ICU CCU Monitoring System (GE Healthcare, Waukesha, WI, USA) (c) and monitor (d). The Galileo ICU ventilator (e). ECMO return (f) and access cannulae (b), respectively. The pump assembly consisting of the non-CE marked air-oxygen mixture (FiO_2_) (Sechrist, Anaheim, CA, USA) (h). CE marked ECMO 550 Bio-Console Bio-Medicus (Medtronic Bio-Medicus Inc., Eden Prairie, MN, USA) (i). CE marked artificial lung (Quadrox PLS) oxygenator (j). Chest drain (K). Sheep (l). Permanent Life Support (PLS) ECMO circuit (MAQUET Cardiopulmonary AG, USA) (m). The Rotaflow Centrifugal Pump and surface coated circuitry, the console pump speed controller (Medtronic) with external drive head, and the heating source are not visible.

The sheep ECMO model is a multifaceted study that was designed to investigate alterations in inflammation and coagulation, drug pharmacokinetics, ECMO induced organ injury, novel echocardiography techniques, and oxidative stress levels during venovenous (VV) ECMO [2, 13–15]. The study also seeks to understand the pathophysiology of ECMO and to determine if the transfusion of aged compared to fresh ovine packed red blood cells (PRBCs) acts to compound ECMO inflammation leading to tissue injury. VV-ECMO is a form of extracorporeal respiratory support, where venous blood is drained from the patient to a gas exchange device (oxygenator) via cannulae placed in a large central vein. Blood then becomes enriched with oxygen, has carbon dioxide removed, and is returned to the right atrium. VV-ECMO is a viable option in critically ill patients with potentially reversible respiratory failure who fail conventional treatment. Ongoing refinements in ECMO technology and clinical delivery may further improve patient outcomes. A typical ECMO animal setup is illustrated in Figure 3.

#### 3.4.2. TRALI Studies

TRALI is considered the most frequent cause of transfusion-related morbidity and mortality; however, the mechanism by which it develops remains to be fully elucidated [76, 125]. Crucial to this is the development of clinically relevant animal models. Thus, our group developed the first large animal model of TRALI, using sheep. In this model, sheep were anaesthetised and instrumented and then infused with* Escherichia coli* lipopolysaccharide (LPS) to simulate bacterial infection, which is one of the risk factors for the development of TRALI. Sheep were then given a 10% transfusion with aliquots of pooled supernatant prepared from date-of-expiry human blood products (either day 5 platelet units or day 42 PRBC units) to provoke the development of TRALI. Control sheep received saline as a control first insult and pooled supernatants from equivalent fresh (day 1) human blood products as a control second insult. In this model, as in clinical cases, TRALI was defined by the development of hypoxaemia (PaO_2_/FiO_2_ < 300 on ABG analysis) and pulmonary oedema (diagnosed by blinded histological assessment of postmortem lung sections), and 80% of LPS-treated sheep that were transfused with the date-of-expiry supernatant developed TRALI. This indicated the importance of both recipient and blood product factors in TRALI, providing further evidence of a two-event model of TRALI pathogenesis. While the incidence of TRALI was identical between these two groups, the physiological changes associated with TRALI induced by PRBC supernatant appeared to be more severe than those induced by the platelet supernatant, with higher PAP and CVP and lower CO. Further studies using the ovine TRALI model are planned to investigate the role that prestorage leucodepletion of blood products may have upon the development of TRALI as well as whether specific protein or lipid factors that form part of the storage lesion of blood products may individually, or in combination, induce TRALI.

#### 3.4.3. Proteogenomic Studies to Understand Selective Susceptibility to Endotoxin

During later TRALI experiments, Chemonges and colleagues [144] observed that “some sheep were more susceptible to the effects of LPS than others from the same mob. As a result, the more susceptible sheep required considerably less LPS to prime their immune system. It is not known why this observation occurred, despite the sheep being the same breed, with similar characteristics. This striking observation of differential susceptibility to LPS has prompted new studies that are seeking to explain the variation” by way of proteogenomic characterisation of circulating acute phase markers and their bioassay development in sheep. This seed study is designed to characterise the selective resistance to the effects of endotoxin; to understand the role of genetic traits, molecular mechanisms, and products associated with the selective susceptibility to endotoxin during priming of the immune system; and to develop comprehensive acute phase protein and circulating microRNA profiles in sheep exposed to endotoxin [144].

#### 3.4.4. CRASH-Sepsis and CRASH-Haemorrhage Studies

These studies are investigating the effects of trauma, haemorrhage, and infection on various tissue beds. In addition, the effects of resuscitation with various intravenous fluids and fresh and stored blood are also being investigated [20]. These models will provide ideal platforms for investigating the interactions between macro- and microcirculation and the effects of various resuscitation targets and techniques on tissue homeostasis. These studies utilise holistic tissue monitoring that includes tissue chemistry analysis, tissue oxygenation, and perfusion measurement using techniques described in earlier sections.

#### 3.4.5. Studies on Acute Smoke Inhalation Lung Injury

**Figure 4:**
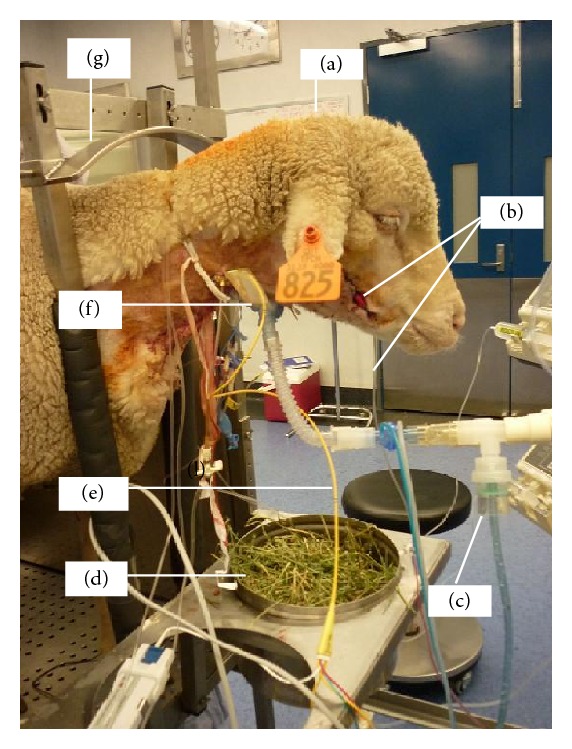
Ovine model of smoke inhalation injury. Nebulisation in a conscious sheep (a) in standing position following smoke inhalation via tracheostomy. Anaesthesia was induced using propofol 4 mg/kg IV and maintained with continuous infusion of midazolam 0.7 mg/kg/hr IV and ketamine 8 mg/kg/hr IV to facilitate instrumentation before allowing the sheep to recover. The right facial artery was cannulated to facilitate continuous arterial blood pressure monitoring and serial arterial blood gas analysis (b). Diphoterine or saline was placed in the nebuliser (c) and the sheep had free access to food (d). A Swan-Ganz pulmonary artery catheter (e) was inserted for continuous monitoring of pulmonary artery pressure, central venous pressure, and continuous cardiac output using thermodilution technique. The animal was ventilated through an open surgical tracheostomy (f). The crush (g) prevented the sheep from making large movements that could dislodge the attached instruments. Animals were monitored for up to 21 hours following instrumentation before being sacrificed.

Pulmonary injury from acute smoke inhalation is a major cause of morbidity and mortality in burns patients [144]. The validated S-ALI model [25] has been very useful in investigating many areas of lung injury and acute respiratory distress syndrome (ARDS) and in understanding the basis of management of lung injury [145]. This model has been used successfully in many experiments such as ovine ECMO and investigations into the management of inhalational lung injury (Figure 4). One of such studies aimed to analyse the effects of nebulising an amphoteric, hypertonic chelating agent for pulmonary decontamination immediately following a standard smoke inhalation injury. It showed a promising treatment strategy for victims of smoke inhalation using an inhalation injury model [25].

#### 3.4.6. The Ovine Brain Stem Death (BSD)

The sheep BSD model provides a tool for the investigation of a wide array of mediators and also the effect of brain stem death on many organs [26, 27]. Brain death causes significant physiologic stress that injures transplantable organs. This reduces the number of transplantable organs, increases posttransplant dysfunction, and contributes to rejection. Large animal models are required to further study pathologic mechanisms contributing to the insult seen in these organs as other current models are of short duration and do not reflect clinical reality. Studies that developed a clinically relevant large animal model of brain death using sheep and investigated the physiological responses that occur over 24 hours have been completed* (unpublished data)*. After initial cannulation and anaesthesia using the techniques described above, an intracranial burr hole was established for insertion of an intracranial pressure monitoring device. For animals randomised to brain death, another cranial burr hole was made and an extradural foley catheter was inserted. This was inflated with 20–30 mL saline to reproduce a rapidly expanding intracranial lesion leading to brain hypoperfusion and brain death. Appropriateness of volume was additionally assessed by further inflation of small aliquots (1 mL) saline into the catheter until the point that no further sympathetic response was obtained. Intracranial pressure was maintained above the mean arterial pressure for 30 min. The criteria used for ascertaining the establishment of BSD were negative cerebral perfusion pressure for 30 min, loss of corneal and papillary reflexes, and no cough on bronchoscopy. Ketamine and alfaxalone were reduced to 2 mg/hr and 1 mg/hr after the diagnosis of brain death. Midazolam was ceased if it had been used. The animals were then maintained for 24 hours at which point they were sacrificed. The sheep received care similar to a human donor, including vasopressors, inotropes, and hormonal therapy [26, 27]. The brain dead animals displayed significant elevations in heart rate, mean arterial pressure, and cardiac index on induction of brain death. Physiologic data has demonstrated that pulmonary pressure significantly increased after brain death and remained elevated for 24 hours. This may contribute to cardiac injury and the right ventricular dysfunction seen after transplantation. This model has been used to investigate the effect of an endothelin antagonist drug on preservation of the cardiopulmonary function following brain death [27]. Studies such as this have the potential to develop techniques to improve the availability and quality of donor organs for transplantation [27].

#### 3.4.7. BiVACOR and BiVAD Artificial Heart and the Ovine Left Ventricular Assist Device (LVAD)

**Figure 5:**
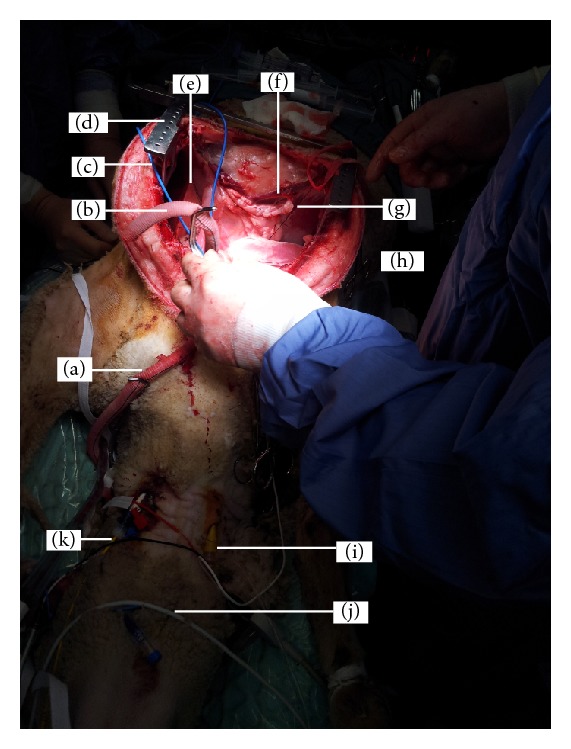
Implanting an artificial heart in an ovine model. This study evaluated a biventricular mechanical device called BiVACOR that is being developed for patients with end stage heart failure. Sheep are anaesthetised and maintained as in humans undergoing cardiac transplantation. In this sheep model, anaesthesia is maintained by constant infusion of propofol and fentanyl. The approach to the heart is via a median sternotomy. Anastomoses to prosthetic blood vessels are created on the pulmonary vein (a) and artery (b) and dorsal aorta and clamped for subsequent attachment to the BiVACOR system (not pictured). In our completed trials, we attached grafts via an end-to-end anastomosis to the ascending aorta (16–18 mm) and pulmonary artery (20 mm) for left and right pump outflows, respectively. Inflow connections were achieved by removing the ventricles just below the atrioventricular groove (leaving about 1 cm of ventricular tissue) and suturing 38 mm grafts (end-to-end) to the ventricular tissue. All grafts were then attached to the pump. Blood flow after the prosthetic heart implant can be monitored by flow meters via probes (c). The surgical exposure of the chest cavity is maintained by a retractor (d) to visualise all organs. The right lung (e), diaphragm, and heart (h) are visible. A stay suture (g) facilitates the suspension of major blood vessels during dissection. Intravenous access for fluid administration was facilitated via a large bore catheter (i). Note the white colour of propofol (j) being used to maintain anaesthesia. During dissection and prior to the removal of the ventricles, continuous haemodynamic monitoring is achieved via the Swan-Ganz pulmonary artery catheter (k).

BiVACOR is a novel rotary total artificial heart which is being developed in part by our group to treat global end-stage heart failure [21–23]. Studies are ongoing and preclinical prototypes have been tested by our group on an ovine model for the artificial heart. Our studies have assessed the haemodynamic performance of the dual pumping device during biventricular assistance (BiVAD) and total artificial heart (TAH) support, whilst assessing the ability to simultaneously alter the left/right heart chamber outflows successfully. We have also assessed the use of dual rotary LVAD systems to provide biventricular support. In these studies, haemodynamic and pump parameters were captured at 100 Hz using a dSPACE acquisition system (DS1103, dSPACE, MI, USA). Systemic and pulmonary flow rates were measured using clamp on perivascular flow sensors (MC20PAU, Transonic Systems, NY, USA) connected to a perivascular flow meter (T402-PP, Transonic Systems, NY, USA). Circulatory and pump pressures were recorded using silicon-based transducers (PX181B-015C5V, Omega Engineering, CT, USA).  Figure 5 shows an anaesthetised sheep with a median sternotomy being prepared for artificial heart implant studies.

#### 3.4.8. Studies to Assess Cerebral Microcirculation

The aim of this model is to describe a novel transseptal catheterisation technique for the injection of colour-coded microspheres for the assessment of cerebral microcirculation [28]. Transseptal catheterization refers to the intracardiac puncture of the interatrial septum with the intention of accessing the left atrium from the right atrium, by a technique originally developed by Ross [146]. The targeted area in a transseptal catheterization is the fossa ovalis (FO), as it is the thinnest region within the septum. Guidance of the procedure as well as identification of the FO is facilitated by fluoroscopy [147] and ultrasonography [148]. After insertion of the transseptal catheter and confirmation of its correct positioning, 4 million colour-coded spheres of 15-micron diameter each are injected hourly (E-Z TRAC; Interactive Medical Technology, Los Angeles, CA) as previously described [149]. Randomly assigned colours are attributed to each injection time in order to minimise selection biases, as previously described [65, 150]. The distribution of the microspheres in brain tissue can then be determined by cytometry in specific laboratories. The novelty of this technique is that cerebral blood flow changes can be assessed over time, as each different colour assigned to a specific time point will be differentiated during count.

## 4. Discussion

This paper is probably the first to document the varied anaesthetic and data collection techniques, monitoring devices, and point-of-care instruments utilised in ovine biomedical research. The disparity in techniques is probably due to study specific requirements, familiarity of use, and availability of equipment in specific research settings. Also, in some reports, little information is published about the monitoring and the fate of the animal model, which leaves information gaps, therefore limiting the understanding of the reproducibility or translatability of those studies. The latter prompted us to present the practices used by our research group, describing animal ICU monitoring practices as a model for ovine biomedical studies. Meticulous monitoring of anaesthetised animals is essential as failure to detect warning signs of impending disaster results in a dead or impaired animal [151, 152].

Our studies are important in that complex and validated ovine models may, to some extent, be comparable to other animal models such as swine that can generate robust mechanistic data that lend themselves to translational research. While each animal model has significant benefits in medical research, none is without weaknesses [153]. There is a dissenting debate that animal-based research is unable to predict human response because animals and humans have different evolutionary trajectories [58]. The alternative view is that animal models should be genetically closer to those of humans to be relevant [59]. Another potential obstacle in the use of sheep in whole animal experiments is that studies that enrol large animals can provoke intense scrutiny from regulatory oversight groups and journal editors, if not meticulously designed to address animal welfare concerns. Current animal ethics requirements emphasise the principle of replacement, refinement, and reduction (3Rs) in research using animals based on established guidelines [154]. Our studies showcase the 3Rs by utilising rational study design that maximises the research opportunities in a given animal without compromising quality, using advances in technology and highly skilled personnel to minimise experimental attrition due to the unexpected loss of experimental animals.

Monitoring devices come in different varieties and can provide misleading information if they do not function correctly or are not properly configured. Artefacts do occur, thus underscoring the necessity for a clinician to physically check the animal first before assuming the instrument is faulty [7]. There is no device that replaces a trained person in monitoring an anaesthetised or even a conscious experimental animal. The studies whose monitoring methods are described here are multifaceted, expensive, and highly refined. They require meticulous planning, animal preparation, anaesthesia induction and maintenance, advanced physiologic monitoring, state-of-the-art data capture, point-of-care technology, and animal aftercare. This level of monitoring has worked successfully at the CCRG Laboratory; however, it may not always be possible to be used elsewhere. Different research establishments have different logistical constraints and perhaps have different goals with their experiments. It comes at a significant cost and is usually only possible in large establishments with appropriate research support and trained personnel. Electronic patient monitors require regular calibration, require software and hardware updating, can rapidly become obsolete, and can represent a significant financial investment. Also, the majority of devices available for monitoring animals were manufactured for use in humans, which may not necessarily be suitable for use in animals. Information on the description of the strengths, weaknesses, and rationale of each monitoring device relating to their use in sheep is still lacking. Moreover, there is no formal standard to act as a benchmark that evaluates the diverse equipment available for use in large animal ICUs. This in turn may influence investigators to only use or recommend the type of equipment available in their particular institution without consideration of the many other options available in the market.

The full use of the available patient monitoring technology together with the extraction and archiving of considerable amounts of physiological data from every experimental animal has positive animal ethics implications. This ultimately minimises the need for repeat experiments by reducing animal numbers used for experiments and archived and reproducible information allows for a more effective transfer of knowledge to others involved in this area of research.

## 5. Conclusions

There are significant alternative approaches to anaesthesia and monitoring of ovine models, probably due to varied experimental goals. Considerable resource investment in technology is necessary to satisfy the needs of monitoring anaesthetised large animals to facilitate the gathering of robust and translatable research data. The experience of the professional and technical personnel equates to the equipment being utilised to their maximum capabilities for cutting edge animal research. Negligible deleterious outcomes when using ovine models are a result of familiarity and using a standardised approach to all aspects of animal care. This level of standard enables the understanding of emerging research tools such as proteogenomics and other new biomedical technologies in a laboratory setting, which highlights the importance of the utilisation of sheep in researching human disease. We have described a critical care setup with multiple ovine models enabling the study of very important aspects for the whole body animal experiments that could provide an alternative to other large animal models such as pigs.

## Figures and Tables

**Table 1:** Validated ovine models of the Critical Care Research Group (CCRG) in Australia.

Model	Total number of sheep required	Publications
Extracorporeal membrane oxygenation (ECMO)	72	[2, 13–16]
Transfusion-related acute lung injury (TRALI)	112	[17–20]
Cardiac mechanical assist device project (CMADP)	9 + (ongoing study)	[21–24]
Colloids, red cells, aged red cells, sepsis, and haemorrhage (CRASH-S and CRASH-H)	16 + (ongoing study)	[20]
Smoke induced acute lung injury (S-ALI)	20	[25]
Brain stem death (BSD)	24	[26, 27]
Intracardiac echocardiography guided transseptal catheterization (ICETSC)	20 + (ongoing study)	[28]

**Table 2:** Technological devices used in anaesthesia and critical care monitoring in biomedical research on ovine models.

Devices	Models
Physiological monitors	Mindray 9200 anaesthetic monitor (Mindray, Shenzhen, China) [31]; multichannel Gould polygraph recorder (Gould, Valley View, OH, USA) [46]; SurgiVet monitor (SurgiVet V9203; Smiths Medical, MA, USA) [30]; Datex monitor (Datex-Engstrom Compact; Datex-Engstrom Inc., Tewksbury, MA, USA) [50]; System 6, (Triton Technology SD, CA, USA) [52]; haemodynamic monitor (MP150, BIOPAC Systems, Goleta, CA, USA) [36]; 7830A (Hewlett Packard, Santa Clara, CA, USA) [35, 41, 44, 45, 54, 56, 65–67, 72–74, 79]; V24C (Philips Medizin Systeme Böblingen, Böblingen, Germany) [53]; IntelliVue MP50 (Philips Medical Systems, Böblingen, Germany) [49]; V24 and V26 (Philips-Agilent, Andover, MA, USA) [68]; and OM9 (Electronics for Medicine, Pleasantville, NY, USA) [40, 53, 68, 75].

Blood gas analysers	iSTAT CG4 (Abbott Laboratories, USA) [31]; Radiometer ABL 700 (Radiometer America Inc., OH, USA) [30]; ABL 700, (Radiometer, Copenhagen, Denmark) [42]; ABL 5 (Radiometer, Copenhagen, Denmark) [69]; ABL 500 (Radiometer, Westlake, OH, USA) [64]; ABL 800 Flex (Radiometer, Copenhagen, Denmark) [37]; IL GEM Premier 3000 (IL GEM, GMI, MN, USA) [33, 43, 48]; 1302 IL (Instrumental Laboratory, Lexington, MA, USA) [38, 68]; IL1600 (Instrumentation Laboratory, Lexington, MA, USA) [39]; and Synthesis 15 (Instrumentation Laboratories, Lexington, MA, USA) [35, 41, 44, 45, 54, 56, 65–67, 72–74, 79].

Pressure transducers	Statham pressure transducer (Statham, Oxnard, CA, USA) [46]; pressure transducers (Edwards Lifesciences LLC, Irvine, CA, USA) [40, 53, 68, 75]; pressure transducer (ITL Healthcare, Australia) [30]; pressure transducer (Medex Medical Ltd., Lancs, UK) [50]; pressure transducer (Vigilance, Edwards Lifesciences, S.A., Saint-Prex, Switzerland) [50]; pressure transducer PT (COBE, Argon, TX, USA) [69]; PX3X3 (Baxter, Edwards Critical Care Division, Irvine, CA, USA) [35, 41, 44, 45, 53, 54, 56, 65, 66, 73, 74, 79]; PX-1800 (Baxter, Edwards Critical Care Division, Irvine, CA, USA) [67, 72]; and PXMK 1590 (Edwards Lifesciences) [49].

Anaesthetic gas analysers	Anaesthetic gas analyser (Datex Capnomac; Datex-Ohmeda, Madison, WI, USA) [46]; Capnomac (Ultima, Datex-Ohmeda, Helsinki, Finland) [61]; lactate + NO (YSI 2300, OH, USA); nitric oxide monitor (Micro Medical, ME, USA) [64, 72]; and GEM Premier 3000 (Instrumentation Laboratory, Lexington, MA, USA) [49].

Blood flow meters	Triton System 6 (model 200, Triton Technology, San Diego, CA, USA) [57, 58]; FBF monitor (model T201/T206, Transonic Systems, Ithaca, NY, USA) [69]; and T208 Transonic Volume Flow Meter (Transonic Systems, Ithaca, NY, USA) [53, 68].

Data acquisition and recorders	PowerLab/8SP (ADInstruments, Castle Hill, Australia) [57, 58]; Data Acquisition System (Cornell University, Ithaca, NY); electronic analog-to-digital converter (NIDAQ, National Instruments, Austin, TX, USA) [69]; and Chart 5 acquisition software (ADInstruments, Sydney, Australia) [60].

Oximeters	OSM3 Haemoximeter, (Radiometer, Westlake, OH, USA) [64]; Haemoximeter (OSM2, Radiometer) [69]; CO-Oximeter IL482 (Instrumentation Laboratory) [35, 41, 44, 45, 54, 56, 65–67, 72–74, 79]; FiO_2_ in-line Oximeter (MiniOx I oxygen analyser, Catalyst Research Corporation, Owings Mills, MD, USA) [53]; and CO-Oximeter 682 (Instrumentation Laboratory, Lexington, MA, USA) [68].

Cardiac output monitors	Vigilance CCO monitor (Edwards Lifesciences, CA, USA) [36, 37]; capnodynamic CO measurement using Fleisch pneumotachograph (Hans Rudolph Inc., Kansas City, MO, USA) [61]; COM-1 (Baxter-Edwards Critical Care Division, Irvine, CA, USA) [35, 41, 44, 45, 54, 56, 65–67, 72–74, 79]; Monitor 9530 (Baxter-Edwards Critical Care, CA, USA) [53]; and 9520A Cardiac Output Computer (American Edwards Laboratories, Irvine, CA, USA) [40, 53, 68, 75].

Brain monitors	EEG (A1000, Aspect Medical Systems, Yatic, MA, USA) [55].

Blood cell counters	WBC (Hemavet, HV950FS, Drew Scientific, Inc., TX, USA) [33, 48].

Protein assay	TP assay refractometer (National Instrument, Baltimore, MD, USA) [39, 75].

Colloid osmometers	4100 (Wescor, Logan, UT, USA) and 4420 (Wescor, Logan, UT, USA) [49].

**Table 3:** Categories of animal intensive care unit (ICU) theatre devices used in ovine models of critical care research at the Critical Care Research Group (CCRG) Laboratory.

Continuous monitors	Life support	Point-of-care technology
Global	(i) Fluid pumps	(i) Blood gas analysis
(i) Cardiovascular (ECG, ABP, HR, CVP, CCO, and SvO_2_)	(ii) Mechanical ventilators	(ii) Centrifuges
(ii) Respiratory oxygenation(SPO_2_), ventilation (ETCO_2_ and EIT)	(iii) Patient warming sources	(iii) Refractometer
(iii) Core body temperature	(iv) Oxygen delivery	(iv) Microdialysate analysers
(iv) Neurological monitoring (cEEG)	(v) Suction apparatus	(v) Multiplatelet function
Regional	(vi) ECMO	(vi) Rotational thromboelastometry
(i) Tissue oximetry	(vii) RRT	(vii) Fluoroscopy
(ii) Tissue perfusion	(viii) HFOV	(viii) Surface and intracardiac echocardiography (USCOM and iCATHe)
(iii) Tissue metabolism	(ix) VADs	(ix) Renal Doppler
	(x) TAH (BiVACOR)	
	(xi) Inhaled NO therapy	

ECG: electrocardiograph; ABP: arterial blood pressure; HR: heart rate; CVP: continuous venous pressure; CCO: continuous cardiac output; SvO_2_: mixed venous oxygen saturation; ETCO_2_: end tidal carbon dioxide partial pressure; EIT: electrical impedance tomography; cEEG: continuous electroencephalogram; ECMO: extracorporeal membrane oxygenation; RRT: renal replacement therapy; HFOV: high frequency oscillatory ventilation; NO: nitric oxide; USCOM: ultrasonic cardiac output monitor; iCATHe: intracatheter echocardiography; VADs: ventricular assist devices; TAH: total artificial heart.
